# Free-water imaging of the nucleus basalis of Meynert in apolipoprotein E4 carriers

**DOI:** 10.3389/fnagi.2025.1597127

**Published:** 2025-09-16

**Authors:** Edward Ofori, B. Blair Braden, Kewei Chen, Yi Su, Richard J. Caselli, Eric M. Reiman

**Affiliations:** ^1^College of Health Solutions, Arizona State University, Phoenix, AZ, United States; ^2^Banner Alzheimer’s Institute, Banner Health, Phoenix, AZ, United States; ^3^Neurology Department, Mayo Clinic, Scottsdale, AZ, United States; ^4^ASU-Banner Neurodegenerative Research Center, Arizona State University, Tempe, AZ, United States

**Keywords:** nucleus basalis of Meynert, cardiovascular risk, apolipoprotein E genotype, free-water imaging, homocysteine

## Abstract

**Introduction:**

The apolipoprotein E (*APOE*) ε4 allele is the strongest genetic risk factor for late-onset Alzheimer’s disease (AD) and cardiovascular disease. This study aimed to investigate the interactive effects of *APOE* ε4 genotype and cardiovascular risk on the microstructure of the nucleus basalis of Meynert (NBM), a key cholinergic region affected early in AD, using advanced diffusion magnetic resonance imaging.

**Methods:**

This cross-sectional study included 167 cognitively unimpaired older adults from the Arizona *APOE* Cohort. Participants were stratified by genotype: *APOE* ε4 non-carriers (*N* = 83), heterozygous carriers (*N* = 51), and homozygous carriers (*N* = 33). Cardiovascular risk was quantified using a composite score calculated by assigning points based on the presence of risk factors (myocardial infarction/peripheral vascular disease, hypertension, diabetes, hypercholesterolemia) and categorized levels of continuous variables (systolic and diastolic blood pressure, body mass index) with higher scores indicating greater risk. Participants underwent comprehensive neuropsychological assessments, structural MRI, diffusion MRI, and Pittsburgh Compound-B (PiB) Positron Emission Tomography imaging.

**Results:**

A significant interaction was found between *APOE* genotype and cardiovascular risk on NBM FW levels (*p* = 0.02). In *APOE* ε3/ε3 and ε3/ε4 carriers, greater cardiovascular risk was associated with increased NBM FW. Conversely, *APOE* ε4/ε4 carriers exhibited similar FW values regardless of their cardiovascular risk category. Furthermore, elevated NBM FW accounted for approximately 25% of the variance in systolic blood pressure, homocysteine, and cholesterol-to-HDL ratio (*p*’s < 0.01). Cardiovascular risk had a more pronounced effect on corrected fractional anisotropy (FA) than on FW measures (*p*’s < 0.05).

**Conclusions:**

The findings suggest that the *APOE* ε4/ε4 accelerates early microstructural alterations within the basal forebrain cholinergic system potentially through mechanisms involving altered lipid homeostasis, compromised neurovascular integrity, and sustained neuroinflammatory responses. These effects appear to indicate a genotype-specific vulnerability. Free-water imaging of the NBM emerges as a sensitive, non-invasive biomarker capable of detecting these *APOE*-modulated microstructural changes before overt atrophy or cognitive decline. Understanding the multifactorial pathways through which *APOE* ε4 and cardiovascular factors confer risk may enable increased understanding in genetically susceptible individuals prior to widespread neurodegeneration.

## Introduction

Alzheimer’s disease (AD) is a progressive neurodegenerative disorder characterized by cognitive decline and memory loss. The *APOE* ε4 allele, the strongest genetic risk factor for AD, plays a key role in lipid metabolism, inflammatory response, and neurovascular function (Caselli et al., 2009; Jackson et al., 2021; Peng et al., 2018; Tzioras et al., 2019). Furthermore, *APOE* ε4 has been associated with faster rates of progression in AD (Kanai et al., 1999; Freudenberg-Hua et al., 2018). It may also act as a modifier of cognitive decline through its interaction with cardiovascular diseases (CVDs) (Scuteri et al., 2005; Wei et al., 2024). The combination of APOE ε4 dosage and CVDs may work synergistically to worsen cognitive outcomes in individuals with both increased genetic and vascular risks (Lee B. C. et al., 2023; Levine et al., 2023).

AD is characterized by early and progressive loss of cholinergic neurons in the NBM, a key nucleus of the basal forebrain (Freudenberg-Hua et al., 2018; Rogers et al., 1985). Furthermore, studies have shown that cholinergic neuron loss in NBM is an important feature of the pathogenesis of neuritic plaque counts, with the *APOE* ε4 allele specifically impairing cholinergic function in animal and neuropathological studies, independent of plaque presence (Arendt et al., 1985; Allen et al., 1997). Conversely, a pathological study has shown sparing of NBM neurons in cases with vascular dementia (Jung et al., 2012). Early detection of *in vivo* changes in the NBM in *APOE* ε4 carriers may provide an understanding of the downstream interaction effects of *APOE* ε4 and cardiovascular risk in aging adults.

The basal forebrain is among the earliest brain regions affected by aging and vascular pathology, even in the absence of overt Alzheimer’s disease. NBM, a specific subregion in the basal forebrain, receives input from the amygdala and hippocampus and also projects to widespread cortical regions (Mihailoff et al., 1989; George et al., 2011; Zaborszky et al., 2015). Notably, the NBM demonstrates selective vulnerability to both cholinergic degeneration and cerebrovascular burden. Subcortical networks involving the NBM may be important in understanding the progression of AD (Shu et al., 2019). Advanced dMRI techniques could aid in elucidating the progressive nature of AD and the neurobiological effect of *APOE* ε4.

Free-water (FW) imaging has been shown to be sensitive to detecting *in vivo* regional microstructural changes in AD (Ofori et al., 2019; Ofori et al., 2024; Archer et al., 2021). The majority of these studies have focused on cortical changes in white matter, and mediotemporal gray matter FW changes have shown promise for detecting changes in the neurodegenerative process (Chu et al., 2022; Maillard et al., 2019; Edde et al., 2020; Montal et al., 2018). FW molecules do not experience bulk flow and are minimally restricted by cellular or extracellular barriers; free water is prone to accumulate in the extracellular space that arises from neuronal injury and neuroinflammatory processes. (Pasternak et al., 2012; Ofori et al., 2015). Early detection may result in a decrease in FW, which would reflect increased cellularity and/or glial recruitment, whereas increases in FW may reflect cellular loss and increased osmotic challenges in the cell (Pasternak et al., 2014; Starck et al., 2021). Most studies in AD populations have found no effect of *APOE* ε4 as a covariate on the FW signal in these regions (Ofori et al., 2019; Chu et al., 2022).

Amyloid-beta (Aβ) and tau pathology have been shown to be elevated in the basal forebrain of AD patients, relative to controls (Mieling et al., 2023; Nakaya et al., 2022). Interestingly, pathology studies have shown an inverse relationship between choline acetyltransferase activity in the temporal cortex with increasing *APOE* ε4 allele copy and an increase in senile plaques and tangles in cortical areas (Beffert and Poirier, 1996). As therapies have been developed to slow the causal influence of AD pathological hallmarks, it is apparent that understanding cholinergic system integrity along with AD pathology may be important for prodromal AD (Ashford, 2015).

Recent inquiries have further delineated the *APOE* ε4 role in modulating inflammatory pathways within the neurodegenerative context (Mieling et al., 2023; Reiman et al., 2024). The *APOE* ε4 gene dose-related brain imaging findings have shown increased amyloid, microglial activation, and translocator protein (TSPO) positron emission tomography (PET) (Reiman et al., 2009; Ferrari-Souza et al., 2023; Fan et al., 2015). Immune modulation in the NBM may occur in response to amyloid-beta or tau pathology; however, it is likely subdetectable with the resolution of most PET studies and may instead be detectable using FW imaging.

*APOE* ε4 impacts cholesterol metabolism, which may influence amyloid-beta and vascular pathogenesis. A recent report suggested that blood-based cardiovascular dysfunction markers are related to brain FW (Mayer et al., 2022; Lee H. et al., 2023); however, it did not examine the effects of *APOE* ε4. The APOE genotype influences blood vessels throughout life, beginning in early development. For instance, an autopsy study found differences in peripheral LDL cholesterol and atherosclerotic plaque formation in vascular structures following a clear pattern, with *APOE* ε4 carriers having the greatest burden of levels (Hixson, 1991). Accordingly, several reports have indicated that *APOE* ε4 carriers have increased odds for cardiovascular diseases (Lahoz et al., 2001; Jofre-Monseny et al., 2008; Weiss et al., 2021).

Collectively, our study aims to investigate the effects of *APOE* ε4 and cardiovascular risk on free-water metrics of the NBM and evaluate relationships with cardiovascular measurements. We hypothesize that homozygous carriers of the *APOE* ε4 gene will have increased FW in the NBM, and this may reflect early neurodegeneration, as shown by studies indicating differences between early mild cognitive impairment (MCI) and cognitively unimpaired. We also suggest that increases in FW would relate to cardiovascular risk measurements in *APOE* ε4 carriers.

## Materials and methods

The present investigation sourced its biological specimens and associated data from the Arizona *APOE* Cohort, a longitudinal endeavor tracking cognitive health across individuals bearing varying quantities of the *APOE* ε4 allele, ranging from none to two copies between 1994 and 2017 (Reiman et al., 2009; Fan et al., 2015). Prior to their participation, all volunteers provided their written informed consent, underscoring the ethical rigor of the study, which received the endorsement of the institutional review boards of both the Mayo Clinic and Banner Health in the surrounding areas of Phoenix.

Evaluative protocols within the study encompassed a comprehensive neurological assessment, alongside a suite of diagnostic tools, including the Folstein Mini-Mental Status Exam (MMSE) for cognitive function (Folstein et al., 1975), and the Hamilton Depression Rating Scale for mood assessment (Hamilton, 1960), Instrumental Activities of Daily Living for evaluating the participants’ ability to perform daily tasks. Furthermore, the Structured Psychiatric Interview for DSM-III-R was used to scrutinize psychiatric conditions, supplemented by a diverse array of neuropsychological examinations and additional clinical evaluations. Importantly, at the inception of the study, none of the participants fulfilled the established diagnostic criteria for amnestic mild cognitive impairment (aMCI), AD, any other dementia variants, or major depressive disorder, ensuring the cognitive integrity and psychological health of the cohort at baseline.

The current analysis included 167 subjects with a mean age of 64.0 ± 7.1 years (range: 49–81) and a mean education level of 16.5 ± 2.0 years. Seventy-five percent (75%) of subjects were women, and 15% were Hispanic. The sample was derived from the initial collection of diffusion imaging data; however, the timing of this collection varied among individuals.

### Magnetic resonance imaging acquisition

Participants underwent structural and dMRI scanning using a 3T GE Discovery 750 scanner at Banner Alzheimer’s Institute between 2013 and 2019. A T1-weighted volumetric inversion-recovery spoiled gradient-recalled echo (IRSPGR) sequence was acquired with the following parameters: echo time (TE) = Min Full, repetition time (TR) optimized per participant, flip angle = 11°, number of excitations (NEXs) = 1, field of view (FOV) = 24 cm, imaging matrix = 256 × 256, and slice thickness = 1.2 mm. For diffusion MRI, an axial 2D echo-planar imaging (EPI) was acquired with TR = 8,400 ms, TE ≈ 85 ms, flip angle = 90°, FOV = 24 cm, and matrix size = 128 × 128 × 59 (covering the whole brain). The voxel size was 1.875 × 1.875 × 2.7 mm^3.^ Diffusion-weighting was applied along 30 non-collinear directions with *b* = 1,000 s/mm^2^, along with 5 b0 images.

## Diffusion MRI analysis

**Figure 1:**
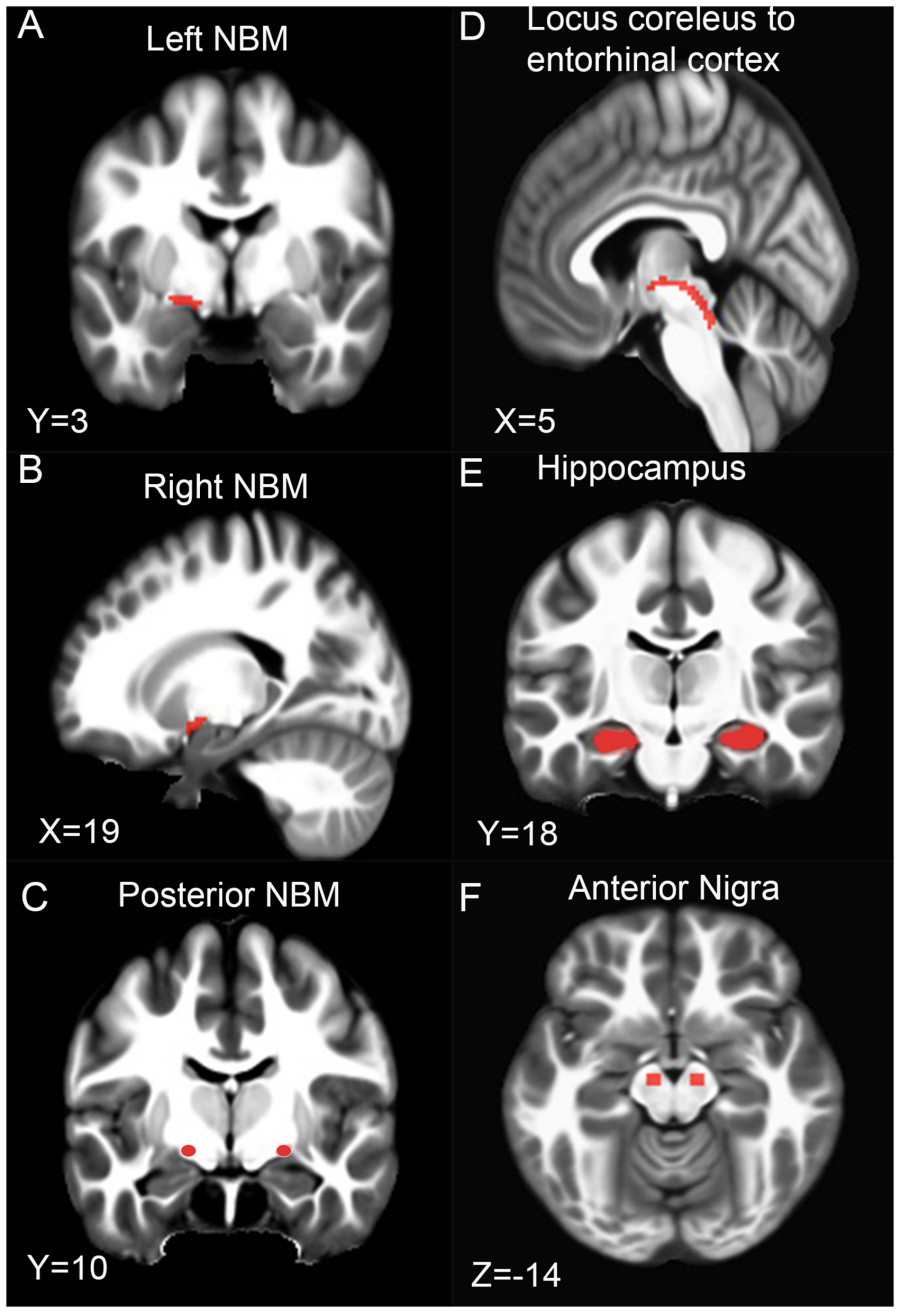
Regions of interest. Regions of interest are shown in Montreal Neurological standard space over a 152 Template for the left NBM **(A)**, right NBM **(B)**, posterior NBM **(C)**, locus to entorhinal cortex tract **(D)**, hippocampus **(E)**, and anterior nigra **(F)**. Total field of view of the diffusion scan covered the entire brain. NBM = nucleus basalis of Meynert. X (Sagittal), Y (coronal), and Z (axial) views are depicted at the corresponding MNI location.

Imaging data from MRI were processed utilizing a custom suite of tools, including the Functional MRI of the Brain (FMRIB) Software Library (FSL), Advanced Normalization Tools, and previously developed UNIX shell scripts (Ofori et al., 2019; Ofori et al., 2015). The pipeline for dMRI processing was fully automated. The correction of scans for distortions induced by eddy currents and head movement was accomplished through affine transformations, with a subsequent adjustment of gradient directions to account for these corrections. Additionally, the elimination of non-brain tissues from the scans was performed. FW is a quantifiable metric derived from dMRI scans, indicating the fractional volume of water that diffuses freely in each voxel. FW maps were generated using a specialized MATLAB script, and the approach adopted for this task aligns with methodologies documented in previous studies (Pasternak et al., 2012; Ofori et al., 2015). Quality control checks were conducted with an eddy current outlier report, visual inspection, and correction for susceptibility. Any motion exceeding 2 mm on average movement, 5 mm on maximal movement, and having > 2% of slice outliers was removed from analysis. The average movement was ~0.9 mm per patient. The spatial distribution of FW across seven predetermined regions (i.e., left and right posterior NBM, left and right NBM, left and right transentorhinal tract to locus coeruleus, and bilateral anterior nigra) of interest (ROIs) is illustrated in Figure 1. Furthermore, diffusion tensors adjusted to negate the partial volume effects of FW were generated, from which fractional anisotropy was calculated using FSL’s DTIFit, resulting in the creation of FW-corrected fractional anisotropy (FA_c_) maps.

The maps for FW in subject space were aligned to the Montreal Neurological Institute (MNI) 152 template space. We computed FA maps in subject space and then warped FA maps to MNI space, 1 mm isotropic FMRIB58 FA template (Jenkinson et al., 2012). An initial 12-d.o.f. affine transform (mutual-information cost) was followed by a non-linear SyN warp. The resulting deformation field was applied to all diffusion-derived maps. To quantify spatial fidelity, we first binarized each subject’s warped FA map and then we then compared this mask with the 50%-thresholded probabilistic NBM from Zaborszky et al. (2008) using *ANTs ImageMath* DiceAndMinDistSum. Across 167 participants, the mean ± SD Dice similarity was 0.88 ± 0.04, and the 95th-percentile Hausdorff distance was 0.43 ± 0.11 mm.

### Centiloid estimation by PET

To harmonize *β*-amyloid Aβ burden quantification, we implemented a Centiloid-standardized analysis pipeline for PiB PET imaging at Banner Alzheimer’s Institute (BAI), drawing upon established Centiloid calibration methods (Klunk et al., 2015). [^11^C]PiB PET images were acquired on GE Discovery PET/CT scanners using 20-min dynamic scans following a 50-min uptake period after intravenous injection of 15 mCi of ^11^C-PiB, with motion correction performed across dynamic frames using rigid-body registration. Resulting motion-corrected PET frames were summed to generate static 50–70 min images and co-registered to high-resolution T1-weighted MRI scans using SPM12, followed by MRI normalization into MNI-152 standard space using SPM8 unified segmentation framework. Standard cortical target ROIs were defined per the Centiloid protocol, comprising prefrontal, orbitofrontal, parietal, lateral temporal, anterior/posterior cingulate, and precuneus cortices, with whole cerebellar crus (WC) serving as the reference region to minimize intersubject variance in SUVr values. Additionally, [^18^F]flortaucipir PET processing followed identical preprocessing steps with 30-min dynamic acquisitions and quantification within bilateral entorhinal and inferior temporal cortices meta-ROI for early tau deposition assessment. Centiloid scaling calibration utilized a two-point anchor approach with data from young cognitively normal controls and typical Alzheimer’s disease cases.

### Basal forebrain regions of interest segmentation

Volumetric MRI measurements were processed using FreeSurfer software (version 7.4.1^1^) (Desikan et al., 2006). Whole basal forebrain volumes [i.e., were segmented and computed using an advanced deep-learning segmentation toolbox (Greve et al., 2021)]. Subregional ROIs within the basal forebrain were derived from a study that correlated histological sections with MNI space (Zaborszky et al., 2008). We used the FreeSurfer ROIs to report volumetric statistics of the basal forebrain (i.e., including Ch1–4), and we used the subregional ROIs for the diffusion analyses. The subregional ROIs were the Ch4 region and posterior NBM to identify microstructural deficits. The tracts linking the locus coeruleus with the transentorhinal cortex were identified using the Brainstem Connectome Atlas, based on connectome imaging data from the Human Connectome Project (Chu et al., 2022; Sun et al., 2020). For each region, mean FW and FA_c_ metrics were computed across each hemisphere of the region.

### Neurodegenerative relevant regions of interest segmentation

We explored the left and right tracts from the locus coeruleus to the entorhinal cortex (LCTEC), the left and right anterior substantia nigra, and the hippocampus (Chu et al., 2022; Planetta et al., 2016). These ROIs were selected based on literature suggesting their involvement in the initial stages of AD and changes in aging and other neurodegenerative diseases. The LCTEC, hippocampus, and nigra ROIs were also based on a standardized mask derived from previous reports (Chu et al., 2022; Ofori et al., 2015; Pagni et al., 2022). These results are included in Supplementary materials.

### Blood panel derivatives

Blood samples were collected from all patients on arrival at Banner Health locations and were fractionated within 2 h of collection and snap frozen in liquid nitrogen. *APOE* genotypes were characterized from venous blood samples (Hixson and Vernier, 1990). Venous samples were acquired, stored at −70° C, and serum lipid profiles characterized to permit the analyses reported here. The serum total cholesterol (TC), CRP high sensitivity, homocysteine, triglyceride, high-density lipoprotein, and low-density lipoprotein (LDL) levels were measured and determined by high-performance gel filtration chromatography (Reiman et al., 2010).

### Cardiovascular risk composite score

A composite cardiovascular risk score was calculated for each participant using a combination of categorical and continuous variables. Categorical variables (myocardial infarction/peripheral vascular disease, hypertension, diabetes, and hypercholesterolemia) were assigned 1 point for ‘Yes’ and 0 points for ‘No’. Continuous variables (systolic blood pressure (BP), diastolic BP, and body mass index [BMI]) were categorized based on clinical guidelines and assigned points accordingly (Whelton et al., 2018; Bakhtiyari et al., 2022) (see Supplementary Table 1). The total scores were summed, and participants were categorized into low (0–3 points), moderate (4–6 points), and high (≥7 points) cardiovascular risk groups.

### Statistical analysis

Means and standard deviations were computed for age, education, BMI, MMSE, Hamilton Anxiety, Beck Depression Inventory, systolic and diastolic BP, UPDRS III total scores, total cholesterol, triglyceride, homocysteine, and cholesterol to HDL ratios. Chi-squared analyses were conducted among *APOE* ε4, sex, and ethnicity. Multivariate ANCOVA was conducted on all volumetric and diffusion-derived ROI measures with *APOE* type (3/3, 3/4, 4/4) and cardiovascular risk as between-subject factors, and age, sex, education, and BMI as covariates for volumetric, FW, and FA_c_. Bonferroni pairwise comparisons were used to examine *post-hoc* effects of significant between-subject factors or interactions for ANCOVA analyses.

We explored linear and non-linear relationships of our dependent significant ROIs from the multivariate ANCOVA with cardiovascular clinical measures using linear regression and *Spearman* correlations within each *APOE* genotype. *p*-values were FDR corrected for correlational analyses.

## Results

### Demographic and cardiovascular risk assessment information

The current analysis included 167 subjects with a mean age of 64.0 ± 7.1 years (range: 49–81) and a mean educational level of 16.5 years. Cognitive and psychological assessments indicate high cognitive functioning (average MMSE score 29.8/30) and low depression levels (average Hamilton Rating Scale for Anxiety score 2.0; Beck Depression Inventory score 4.4). Motor function shows minimal impairment (average UPDRS-III score 0.1, see Table 1). We also examined changes across demographic and cardiovascular risk information within the cohort. The current analysis also revealed an average BMI of 27.2 and average BP readings of 130/75 mmHg. Lipid profiles showed an average total cholesterol of 184 mg/dL, variable triglyceride levels (average 113.2 mg/dL), and a favorable cholesterol/HDL ratio of ~3.2. The average for hsCRP 1.83 mg/L and metabolic average homocysteine 9.5 μmol/L markers was slightly elevated (see Table 1). We also computed the information across *APOE* genotype and only found significant differences in CRP High Sensitivity levels and cholesterol (See Supplementary Table 2).

**Table 1:** Demographic, clinical, and cardiovascular information.

Participant information	Statistics (*N*=167)^1^
ApoE 3/3|3/4|4/4	83|51|33
Age	64.0 (7.1)
Sex, % Female	75%
Ethnicity, % non-Hispanic	86%
Education	16.5 (2.0)
Cardiovascular risk (Low/Moderate/High)	71/57/20
MMSE	29.8 (0.6)
Hamilton	2.0 (2.7)
Beck depression inventory	4.4 (4.3)
UPRDS-III total score	0.1 (0.6)
BMI (kg/m^2^)	27.2 (5.6)
Blood pressure in mmHg(Systolic/Diastolic)	130 (19.8) / 75 (11.1)
Total cholesterol in mg/dL	184.0 (32.5)
CRP high sensitivity in mg/L	1.83 (2.0)
Homocysteine in mcmol/L	9.5 (2.6)
Triglyceride in mg/dL	113.2 (61.1)
Cholesterol to high-density lipoprotein	3.2 (1.0)
Estimated intracranial volume (L)	1.43 ± 0.19
Centiloid (*n* = 65)	14 (21)

^1^ n/N (%); mean (SD), BMI = body mass index, CRP (C-reactive protein), dL = deciliter, Hg = mercury, kg = kilograms, L = liter, m = meters, mcmol = micromolar, mg = milligrams, MMSE = Mini-Mental State Exam.

### Multivariate ANCOVA: interaction of *APOE* and cardiovascular risk results for NBM ROIs

**Figure 2:**
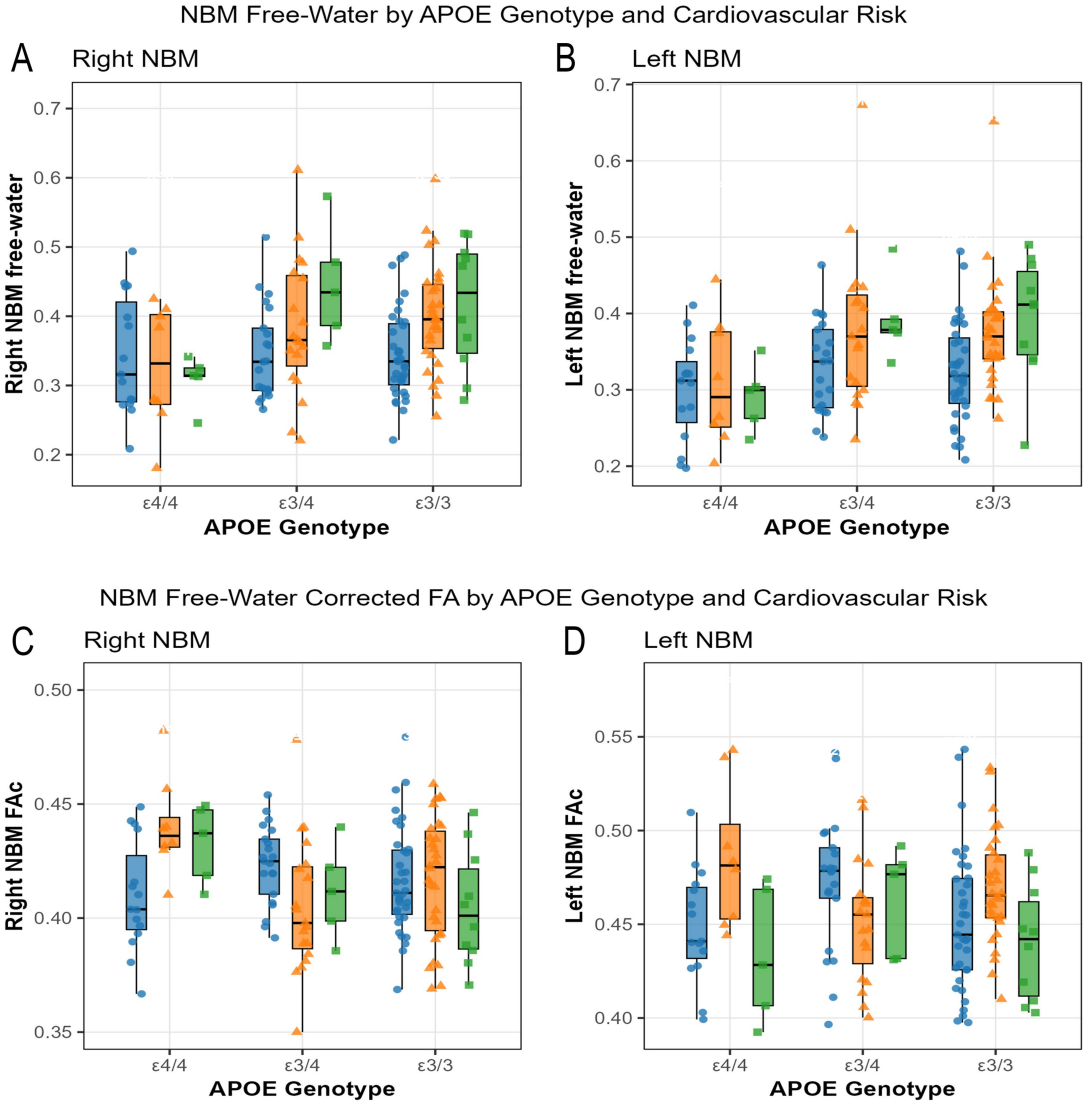
Increased cardiovascular risk decreases NBM FW in *APOE* ε4/4 carriers Free-water values are shown for right **(A)** and left **(B)** NBM by APOE genotype (*x*-axis) and cardiovascular risk tertile. Free-water corrected FA values are shown for the right **(C)** and left **(D)** NBM, similar to the free-water plot. Each dot represents one participant; boxes show median with interquartile range (IQR); whiskers extend to 1.5 × IQR. Color and shape coding: blue circles = low cardiovascular risk; orange triangles = moderate cardiovascular risk; green squares = high cardiovascular risk. NBM = nucleus basalis of Meynert; IQR = interquartile range.

Our results revealed a significant interaction of *APOE* and cardiovascular risk on right NBM free-water [(*F*(4, 136) = 3.0, *p* = 0.022, η_p_^2^ = 0.080)] and left NBM free-water values[(*F*(4, 136) = 2.9, *p* = 0.026, η_p_^2^ = 0.078)]. The interaction revealed that the high cardiovascular risk category resulted in decreased free-water for *APOE* ε4/4, whereas the high cardiovascular risk category resulted in increased free-water for *APOE* ε3/4 and *APOE* ε3/3 carriers. Left NBM FW interaction resulted in increased free-water in *APOE* ε3/4 carriers with high cardiovascular risk when compared to all other conditions (see Figure 2). Specifically, among *APOE* ε3/4 carriers, individuals with high risk exhibited significantly elevated FW in right NBM (mean difference = 0.102, SE = 0.036, *p* = 0.016, 95% CI [0.015, 0.189]) compared to those with low risk. Among *APOE* ε4/4 carriers, left NBM FW differed significantly between high and moderate risk (mean difference = 0.049, SE = 0.018, *p* = 0.025, 95% CI [0.005, 0.093]). In other words, within the *APOE* genotype, *APOE* ε4/4 carriers seem to have similar FW values across cardiovascular risk categories, whereas in *APOE* ε3/4 and *APOE* ε3/3 carriers, FW increased with increasing cardiovascular risk.

Our results indicated a significant interaction effect for left and right NBM corrected FA values (*p*’s < 0.05). *APOE* ε4/4 carriers had increased FA_c_ values in moderate and high risk when compared to low risk for the right NBM. For the left NBM region, *APOE* ε4 carriers with the highest risk had the lowest FA_c_ values; whereas for *APOE* ε3/4 carriers, moderate risk FA_c_ values were less than lower risk FA_c_ values. In the right NBM region, *APOE* ε4/4 carriers with moderate cardiovascular risk exhibited lower FA compared to those with low risk (mean difference = −0.031, SE = 0.011, *p* = 0.012, 95% CI [−0.057, −0.005]). In the left NBM, *APOE* ε4/4 high-risk individuals had significantly higher FA than moderate-risk individuals (mean difference = 0.049, SE = 0.018, *p* = 0.025, 95% CI [0.005, 0.093]). In *APOE* ε4/4 carriers, lower FA was observed in moderate- vs. low-risk individuals for the left NBM (mean difference = −0.028, SE = 0.011, *p* = 0.028, 95% CI [−0.054, −0.002]). No significant interactions were found between basal forebrain volumetric and centiloid units (*p*’s > 0.05).

### Multivariate ANCOVA: *APOE* effect results for NBM ROIs

Our results indicated a significant effect on left and right NBM (*p*’s < 0.05). *APOE* ε4/4 carriers had lower free-water for the left NBM [*F*(2, 136) = 8.222, *p* = 4 × 10^−4^, η_p_^2^ = 0.108] and right NBM [*F*(2, 136) = 6.884, *p* = 0.001, η_p_^2^ = 0.092] (see Figure 2 and Supplementary Table 3A). No effects for the covariates of sex and education. A covariate effect for age was shown for all the regions of interest (*p*’s < 0.05, η_p_^2^’s > 0.04). There were no significant effects on the other regions of interest (*p*’s > 0.05). There were no significant effects for *APOE* on FA_c_ measures (*p*’s > 0.05; see Supplementary Table 3B).

There was a significant effect of APOE e4 on centiloid values. Bonferroni comparison revealed that *APOE* ε4/4 had greater centiloid values (41.5) than *APOE* ε3/3 carriers (7.6). No significant effects were observed on left or right basal forebrain volume [*F*(2,136) = 0.25, *p* = 0.78].

### Multivariate ANCOVA: cardiovascular effect results for NBM ROIs

There were no significant effects of cardiovascular risk on centiloid, basal forebrain volumetric, and free-water measures across regions [*F*’s < 2.9, *p*’s > 0.05]. There were significant effects of cardiovascular risk on corrected FA measures. Left NBM [(*F*(2, 136) = 3.36, *p* = 0.038, η_p_^2^ = 0.047)] and left posterior NBM [(*F*(2, 136) = 4.104, *p* = 0.019, η_p_^2^ = 0.057)] revealed significance. Generally, higher cardiovascular risk resulted in lower FA_c_ values in NBM.

### The association among free-water values, blood panel markers, and *APOE*

**Figure 3:**
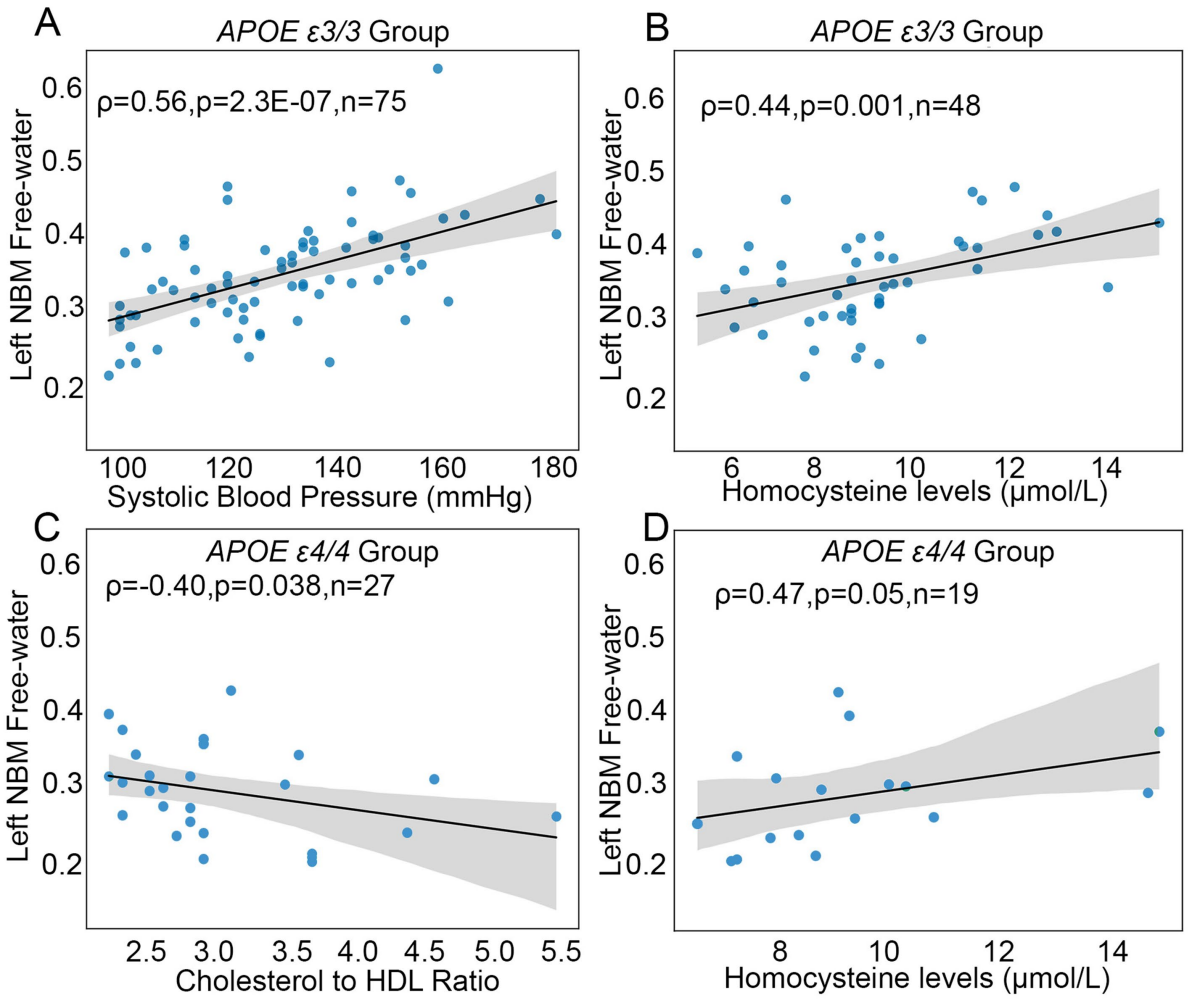
NBM FW correlates with cardiovascular laboratory measures. Figure 3 illustrates scatterplots showing the significant Spearman rho correlations between left nucleus basalis of Meynert (NBM) free-water (FW) values (on the y-axes), blood pressure (on the *x*-axis, **A**) homocysteine (on the *x*-axis, **B,D**), and cholesterol to HDL ratio (on the *x*-axis, **C**) in homozygous *APOE* ε3/3 **(A,B)** and *APOE* ε4 carriers **(C,D)**, NBM = nucleus basalis of Meynert, HDL = high density lipid protein. Shaded 95% confidence intervals with linear fit lines were placed on each sample for association.

For *APOE* ε4 non-carriers, right and left NBM FW were positively associated with systolic BP [*ρ*(75) = 0.47, *p* < 0.01; *ρ*(75) = 0.56, *p* < 0.01; Figure 3A], cholesterol to HDL ratios [*ρ*(74) = 0.33, *p* < 0.01; ρ(74) = 0.24, *p* < 0.05], and homocysteine [ρ(48) = 0.37, *p* = 0.01; ρ(48) = 0.45, *p* < 0.01; Figure 3B]. Left NBM FW was positively associated with diastolic BP [ρ(75) = 0.25, *p* < 0.05; see Supplementary Tables 4–7]. For *APOE* ε4/4 carriers, left NBM FW was positively associated with homocysteine (ρ = 0.48, *p* = 0.05) and negatively associated with cholesterol to HDL ratios (ρ = 0.40, *p* = 0.039) (see Figure 3C and D). For *APOE* ε3/4 carriers, left and right NBM were positively associated with systolic BP [ρ(45) = 0.38, *p* = 0.01; ρ(45) = 0.41, *p* < 0.01; see Supplementary Tables 4–7]. There were no significant correlations observed among centiloid vs. NBM FW measures, centiloid vs. blood panel markers, and volumetric ROIs vs. blood panel markers.

## Discussion

We sought to investigate whether free-water imaging could detect differences in the NBM across varying *APOE* genotypes. It has been well established that neuronal loss within the cholinergic NBM cascades events that lead to mediotemporal lobe degeneration (Ofori et al., 2015). Our findings indicated decreased NBM FW values in *APOE* ε4/4 carriers when compared to *APOE* ε3/4 and non-carriers. We also found significant associations of increased NBM FW values with increased homocysteine levels and systolic BP for *APOE* ε3/3 carriers. In *APOE* ε4/4 carriers, a unique association of increased NBM FW values with decreased cholesterol to HDL ratios indicates that *APOE* may interact with NBM to modulate lipid metabolism. We posit that decreased NBM FW in *APOE* ε4/4 may reflect that *APOE* ε4/4 increases cellularity in the NBM through altered neurovascular and neuroinflammatory processes in comparison to non-carriers of *APOE* ε4. Our findings have various implications for how FW imaging may be useful for aging, genetic influences on the brain, and AD research.

Previous studies have indicated that *APOE* ε4 exacerbates neuronal loss in the NBM, while other studies have not, as this may be due to differences in the ability to quantify neuronal loss (Zhao et al., 2020; Lai et al., 2021). Furthermore, the association between *APOE* ε4 and neurodegeneration may be due to its accelerated effects through either hypometabolism, amyloid, or tau (Lerch et al., 2022; Snellman et al., 2023). Our findings are in line with others that *APOE* ε4 carriers show altered brain characteristics compared to non-carriers (Reiman et al., 2009). We did not observe FW changes in the LC to the entorhinal cortex tracts, where tau pathology is suggested to begin, and would result in FW changes that have been seen in other reports (Chu et al., 2022). One difference between the current report and previous investigations is the cognitive status of participants. Other studies have compared cross-sectional data across impaired vs. intact cognitive status, when presumably the pathological process has been occurring (Ofori et al., 2019). Our cohort has no cognitive impairment and a cohort mean age of 64 years vs. 71 years or older in previous studies. Furthermore, previous studies covaried for *APOE* ε4 or combined heterozygous and homozygous carriers in the *APOE* ε4 group. We were able to evaluate dose effects in regions potentially susceptible to AD and found differences only in the NBM.

Our findings are in line with previous findings suggesting that FW may reflect downstream consequences of hemodynamic alterations (Rydhög et al., 2017; Edwards et al., 2024). We found that higher NBM FW levels were associated with increases in systolic BP in non-carriers and *APOE* ε3/4. A previous report from the Framingham Heart study in over 2000 adults with a mean age of ~55 showed that systolic BP has a direct influence on cerebral FW white matter, and this is moderated by carotid pulse wave velocity (Maillard et al., 2017). We extend these findings to suggest that hemodynamic alterations may also influence gray matter FW values in *APOE* ε4 non-carriers. We did not find a significant association between NBM FW and systolic BP in *APOE* ε4/4 carriers. It has been suggested that *APOE* ε4 and BP interact to increase the risk of small vessel disease (Mayer et al., 2022; Walker et al., 2017; Biffi et al., 2019). *APOE* plays a critical role in determining the risk and severity of cerebral amyloid angiopathy, which is characterized by the accumulation of amyloid-beta. One report found a correlation between white matter FW and diastolic BP when controlling for various demographic and lifestyle variables in individuals with metabolic syndrome (Andica et al., 2023). The lack of correlation in only *APOE* ε4 carriers may be due to a multitude of factors, and *APOE* ε3 may play a key role in modifying BP through the interaction with the hypothalamus (Carmichael and Wainford, 2015).

The novel finding of increased homocysteine levels positively related to increased NBM FW levels across *APOE* ε4/4 and non-carriers suggests that increased free-water levels may reflect neurovascular pathology. Elevated homocysteine levels, a risk factor for cardiovascular disease, have been observed in individuals carrying the *APOE* ε4 allele. Homocysteine is known to contribute to endothelial dysfunction and atherosclerosis. Our findings are similar to another study that showed increased cerebral WM FW was positively associated with increased cardiovascular health markers (Ji et al., 2023). One difference between the current study and Ji et al. is that the association was found in our cognitively normal participants, whereas significant correlations in the Ji et al. study were only found in individuals experiencing some form of cognitive impairment (Ji et al., 2023). Our findings are also in line with findings showing that elevated homocysteine is associated with increased atrophy in the medial temporal lobe in aMCI (Ma et al., 2022). There is growing evidence that homocysteine levels are related to cognitive impairment, as individuals with AD have greater vitamin B deficiencies. Our findings suggest that NBM FW values and homocysteine may provide insight into the longitudinal effects on cognition.

Decreased NBM FW could reflect increased lipid metabolism through increasing extracellular cholesterol in *APOE* ε4 carriers. Cholesterol can control the levels and dynamics of cholinergic neurons and modulates their diffusion. Increased brain cholesterol has been suggested to occur due to amyloid buildup (Piccarducci et al., 2023). Our findings of decreased extracellular space levels with increased cholesterol to HDL ratios are in line with this potential mechanism. It has been shown that myelin breakdown releases excess cholesterol into the extracellular space. One report suggests that plasma 24-hydroxycholesterol (24-OHC) may be a sensitive marker of increased cholesterol metabolism during acute demyelination early in the neurodegenerative processes prior to neuron loss (Hughes et al., 2012). Although we do not have measures of brain cholesterol, perhaps future studies examining the interconnectedness between brain and peripheral lipid metabolism and free water may help unravel how cholesterol metabolism is affecting NBM FW.

The exact pathophysiological mechanism behind the accumulation of brain extracellular water remains unclear. One of the main advantages of diffusion imaging is that it provides microstructural information that can be related to processes within the tissue. Current research has not definitively determined whether the increase in extracellular water is due to neurodegenerative processes, vascular pathology, or a combination of both. In the context of neurodegenerative diseases, increases in FW may reflect microstructural damage and increased CSF in the region, whereas decreases in FW may reflect gliosis or cellularity (Dubelaar et al., 2004). Recent studies have demonstrated that amyloid-beta interacts with cortical tau pathology to influence neurodegeneration. Aβ toxicity is expected to cause inflammatory changes detectable through FW imaging. Furthermore, FW has been found to be correlated with plasma Aβ42/40 in PET-negative participants without dementia, although these patients had altered cognitive status (DeSimone et al., 2024). Our findings showed a decrease in cognitively normal *APOE* ε4 carriers. This may reflect a critical window prior to tau involvement that could impact NBM microstructural environments. Interestingly, FA differences were not uniformly lower in higher cardiovascular risk groups, suggesting that FA may reflect both axonal degeneration and compensatory remodeling depending on APOE genotype and regional vulnerability. Our NBM findings are synergistic with the lack of FW or FA_c_ differences in the locus coeruleus to entorhinal tracts. Given the accumulation of studies showing elevated FW changes when presumably the pathophysiological processes have occurred, our findings may suggest that increases in FW reflect potential influences of tau accumulation, and where FW decreases may reflect increased glial cellularity. The increase in glial cells is likely part of a neuroinflammatory response, as it has been shown that *APOE* ε4/4 may recruit these cells at an enhanced rate. An increase in the number and size of glial cells would decrease the extracellular volume (that is, decreased FW measure). Interestingly, there are few studies examining the effects of disease-related genetic factors on free-water imaging. Further studies would be required to determine whether these differences are related to long-term symptom changes.

We identified a left-lateralized association between HDL-to-cholesterol ratios and NBM fractional water. Previous tracing and neuroimaging studies have demonstrated that the NBM exhibits denser projections to limbic and association cortices in the left hemisphere (Zaborszky et al., 2015). This anatomical asymmetry may potentially explain the observed left-hemispheric sensitivity to lipid dysregulation. Furthermore, a recent [^11^C]PiB PET study, cognitively normal *APOE* ε4/4 carriers showed greater left-than-right cortical amyloid burden (Kjeldsen et al., 2022). Most reports typically average the right and left regions. The study also indicated that *APOE* ε4 does not appear to bias the spatial distribution of pathology toward each hemisphere (Kjeldsen et al., 2022). Additional research is necessary to fully understand the mechanisms underlying lateralized neurodegeneration in individuals with the *APOE* ε4.

Importantly, the use of single-shell imaging to compute free-water has been criticized, given its constrained ability to tease apart partial volume effects from true microstructural alterations. Nonetheless, our observation of a measurable FW decrease in cognitively normal *APOE* ε4 carriers shows that single-shell models can still detect physiologically relevant variations. The primary benefit of single-shell approaches is their feasibility in retrospective or multi-site studies, where the availability of multi-shell data may be limited. However, because single-shell methods may conflate different diffusion compartments, the precision of FW estimations can be adversely impacted and tissue measures underestimated. Replicating these findings with multi-shell acquisitions or complementary techniques would provide more rigorous confirmation of the underlying neurobiological events. More studies also showing the adverse impact of FW and corrected measures diffusion metrics on gray matter and vascular-rich areas are needed.

Our study has some limitations, as there was a small number of *APOE* ε4/4 carriers that had peripheral measurements compared to *APOE* ε3/4 and non-carriers, limiting the statistical power of our correlations. Furthermore, we did not have inclusion of *APOE* ε2 carriers, which may also limit the generalizability of our findings. *APOE* ε2 has been associated with potential neuroprotective effects, including reduced amyloid burden and possibly altered inflammatory responses, which could modulate FW measurements. The lack of full PET characterization in all subjects limits the generalizability of all APOE ε4/4 carriers. Initial diffusion metrics were collected at differing times throughout enrollment, and this may have some influence on the findings we observed. Lack of ethnic and racial diversity, over-representation of women, lack of integrated information on menopause status or hormonal use, and a cross-sectional approach that limits insights into the onset of differences between *APOE* groups. Future investigations should aim to link NBM FW, cardiovascular, and inflammatory marker values with cognitive aging trajectories to determine the functional influences of *APOE*-related allelic differences.

## Conclusion

This is the first investigation to characterize the effect of *APOE* ε4 on FW imaging in cognitively unimpaired late middle-aged older adults. We reveal that *APOE* ε4/4 carriers have decreased NBM FW levels in comparison to *APOE* ε3/4 and non-carriers, whereas no differences were found in the locus coeruleus to entorhinal track. *APOE* ε4/4 also had an inverse relation between increased free-water and an elevated cholesterol/HDL ratio, perhaps suggesting that elevated cholesterol levels impinge on the NBM extracellular space. Furthermore, NBM FW levels were related to peripheral markers of homocysteine and systolic BP, suggesting that FW may also reflect downstream biomechanical and chemical modulators of vascular dysfunction. These differences may reflect modulatory inflammatory processes in *APOE* ε4 carriers, as FW techniques are optimal to estimate neuroinflammation with MRI. The involvement of the *APOE* ε4 allele in modulating these processes, particularly its impact on the NBM and its potential role in AD pathogenesis, warrants further investigation. Elucidating the complex interplay between genetic, vascular, and inflammatory factors may provide valuable insights into the mechanisms underlying cognitive impairment and neurodegenerative diseases, ultimately informing the development of targeted therapeutic strategies.

## Data Availability

The datasets presented in this study can be found in online repositories. The names of the repository/repositories and accession number(s) can be found below: Arizona Alzheimer Consortium.

## Footnotes

^1^ https://surfer.nmr.mgh.harvard.edu
