# Supplementary material for: Free-water imaging of the nucleus basalis of Meynert in apolipoprotein E4 carriers
Source: Front Aging Neurosci. 2025 Sep 16;17:1597127. doi: 10.3389/fnagi.2025.1597127 (PMC12479503; doi:10.3389/fnagi.2025.1597127)
Supplement: Supplementary file 1 [file Table_1.docx]

Supplementary Material

Supplementary Table 1. Categorization of Continuous Variables for Composite Cardiovascular Risk Score

| Variable | Criteria | Points Assigned |
| --- | --- | --- |
| Systolic Blood Pressure (mmHg) | < 120 | 0 |
|  | 120–129 | 1 |
|  | 130–139 | 2 |
|  | ≥ 140 | 3 |
| Diastolic Blood Pressure (mmHg) | < 80 | 0 |
|  | 80–89 | 1 |
|  | ≥ 90 | 2 |
| Body Mass Index (kg/m²) | < 25 | 0 |
|  | 25.0–29.9 | 1 |
|  | 30.0–34.9 | 2 |
|  | 35.0–39.9 | 3 |
|  | ≥ 40 | 4 |

Supplementary Table 2. Demographic, Clinical, and Cardiovascular Information by *APOE* Genotype

|  | *APOE ε3/3* | *APOE ε3/4* | *APOE ε4/4* | Statistical Measures |
| --- | --- | --- | --- | --- |
| N | 83 | 51 | 33 |  |
| Age | 63.9 (6.9) | 64.7 (7.7) | 63.2 (6.8) | F(2,164)=0.49,p=0.61 |
| Sex, % Female | 77% | 70% | 78% | Χ^2^(2,N=167)=0.9,p=0.6 |
| Ethnicity, % non-Hispanic | 79% | 86% | 100% | Χ^2^(2,N=167)=8.1,p=0.02 |
| Education | 16.6 (1.8) | 16.4 (2.1) | 16.5 (2.2) | F(2,164)=0.21,p=0.81 |
| Cardiovascular Risk (Low/Moderate/High) | 39/34/10 | 24/21/6 | 17/9/7 | Χ^2^(4,N=167)=3.1,p=0.55 |
| MMSE | 29.8 (0.4) | 29.7 (0.6) | 29.6 (0.8) | F(2,164)=1.05,p=0.37 |
| Hamilton | 1.7 (2.1) | 2.4 (3.5) | 1.9 (2.6) | F(2,164)=1.18,p=0.31 |
| Beck Depression Inventory | 3.7 (3.4) | 5.4 (5.2) | 4.6 (4.5) | F(2,164)=2.72,p=0.07 |
| UPRDS-III Total Score | 0.13 (0.8) | 0.0 (0.0) | 0.0 (0.0) | F(2,164)=1.04,p=0.35 |
| BMI (kg/m^2^) | 27.3 (5.1) | 27.3 (6.3) | 26.6 (5.6) | F(2,164)=0.20,p=0.82 |
| Systolic Blood pressure in mmHg | 128 (19.8) | 130 (19.7) | 132 (19.8) | F(2,164)=0.47,p=0.63 |
| Diastolic Blood pressure in mmHG | 76.5 (9.9) | 74.6 (9.4) | 76 (15.6) | F(2,164)=0.50,p=0.61 |
| Total Cholesterol in mg/dL | 192.7 (30.8) | 175.5 (33.1) | 174.3 (30.4) | F(2,162)=6.4,p=0.002 |
| CRP High Sensitivity (mg/L) | 2.1 (2.2) | 2.1 (2.0) | 0.69 (0.5) | F(2,108)=4.7,p=0.011 |
| Homocysteine in mcmol/L | 9.5 (2.7) | 9.4 (2.6) | 9.5 (2.4) | F(2,108)=0.05,p=0.95 |
| Triglyceride in mg/dL | 115(60) | 110(60) | 112(66) | F(2,162)=0.14,p=0.87 |
| Cholesterol to High-density lipoprotein | 3.3 (1.1) | 3.1 (0.86) | 3.0 (0.8) | F(2,162)=1.55,p=0.21 |
| Total Intracranial Volume (L) | 1.4±0.20 | 1.4±0.20 | 1.4±0.20 | F(2,162)=0.294,p=0.75 |
| Centiloid | 9.7±15 | 18.1±32 | 24.3±20 | F(2,60)=2.35,p=0.10 |

Supplementary Table 3A & 3B Tables. Means and standard deviations for free-water and fractional anisotropy corrected for free-water

1. Mean and standard deviations for ROIs of free-water

|  | *APOE* ε3/3 | *APOE* ε3/4 | *APOE* ε4/4 |
| --- | --- | --- | --- |
| Left Basal Forebrain Volume (% ICV) | 0.02 (0.003) | 0.02 (0.004) | 0.02 (0.003) |
| Right Basal Forebrain Volume (% ICV) | 0.02 (0.002) | 0.02 (0.003) | 0.02 (0.003) |
| Left NBM | 0.35 (0.07) | 0.35 (0.08) | 0.30 (0.07) |
| Right NBM | 0.38 (0.08) | 0.37 (0.09) | 0.33 (0.08) |
| Left posterior NBM | 0.28 (0.06) | 0.29 (0.05) | 0.28 (0.08) |
| Right posterior NBM | 0.24 (0.04) | 0.24 (0.04) | 0.24 (0.05) |
| L Anterior SN | 0.11 (0.04) | 0.10 (0.03) | 0.10 (0.02) |
| R Anterior SN | 0.13 (0.04) | 0.13 (0.05) | 0.12 (0.04) |
| Left LC to Entorhinal Cortex | 0.18 (0.03) | 0.19 (0.03) | 0.18 (0.03) |
| Right LC to Entorhinal Cortex | 0.19 (0.03) | 0.20 (0.03) | 0.19 (0.02) |
| Left Hippocampus | 0.20 (0.01) | 0.22 (0.01) | 0.20 (0.01) |
| Right Hippocampus | 0.21 (0.01) | 0.22 (0.01) | 0.20 (0.01) |

L= Left, LC=locus coeruleus, NBM=nucleus basalis of Meynert, SN=substantia nigra

1. Mean and standard deviations for ROIs of corrected Fractional Anisotropy

|  | *APOE* ε3/3 | *APOE* ε3/4 | *APOE* ε4/4 |
| --- | --- | --- | --- |
| Left NBM | 0.46 (0.03) | 0.46 (0.03) | 0.46 (0.04) |
| Right NBM | 0.42 (0.03) | 0.41 (0.02) | 0.42 (0.03) |
| Left posterior NBM | 0.38 (0.05) | 0.39 (0.05) | 0.39 (0.06) |
| Right posterior NBM | 0.37 (0.04) | 0.36 (0.04) | 0.38 (0.04) |
| L Anterior SN | 0.61 (0.10) | 0.60 (0.09) | 0.62 (0.07) |
| R Anterior SN | 0.59 (0.08) | 0.60 (0.08) | 0.61 (0.08) |
| Left LC to Entorhinal Cortex | 0.45 (0.03) | 0.45 (0.03) | 0.46 (0.02) |
| Right LC to Entorhinal Cortex | 0.44 (0.02) | 0.44 (0.02) | 0.45 (0.02) |
| Left Hippocampus | 0.34 (0.01) | 0.33 (0.01) | 0.32 (0.01) |
| Right Hippocampus | 0.38 (0.004) | 0.38 (0.01) | 0.37 (0.01) |

L= Left, LC=locus coeruleus, NBM=nucleus basalis of Meynert, SN=substantia nigra

Supplementary Table 4 Table. Spearman rho correlations among left and right nucleus basalis of Meynert free-water levels cardiovascular lab panel measures, and blood pressure measurements across all genotype.

|  | Cardiovascular Lab Panel Measures & Blood Pressure Measurements | | | | | | |
| --- | --- | --- | --- | --- | --- | --- | --- |
| Region of Interest | Systolic  BP (N=147) | Diastolic  BP (N=148) | Triglycerides (N=144) | Cholesterol/HDL Ratio (N=144) | Homocysteine (N=93) | CRP High Sensitivity(N=93) | Cholesterol (N=148) |
| Left NBM  FW | **ρ=0.43,p=4.9 x10^-8^** | **ρ=0.21,p=0.01** | ρ=-0.14,p=0.50 | **ρ=-0.40,p=0.04** | **ρ=0.27p=0.01** | ρ=-0.04,p=0.70 | ρ=-0.04,p=0.70 |
| Right NBM  FW | **ρ=0.36,p=0.55x10^-6^** | ρ=0.13,p=0.51 | ρ=0.13,p=0.51 | ρ=0.23,p=0.25 | **ρ=0.26,p=0.01** | ρ=0.01,p=0.93 | ρ=-0.07,p=0.42 |

BP=Blood Pressure; CRP=C-reactive protein; FW=free-water; HDL=High Density Lipids; NBM=Nucleus of Basal Meynert; Bold values indicate significance at the p<0.05. Units of measurement for BP in mmHg, Triglycerides in mg/dL, homocysteine (mcmol/L), CRP Sensitivity in mg/L and cholesterol in mg/dL.

Supplementary Table 5 Table. Spearman rho correlations among left and right nucleus basalis of Meynert free-water levels, cardiovascular lab panel measures, and blood pressure measurements for *APOE* ε3 homozygous carriers.

|  | Cardiovascular Lab Panel Measures & Blood Pressure Measurements | | | | | | |
| --- | --- | --- | --- | --- | --- | --- | --- |
| Region of Interest | Systolic  BP (N=75) | Diastolic  BP (N=75) | Triglycerides (N=74) | Cholesterol/HDL Ratio (N=74) | Homocysteine (N=48) | CRP High Sensitivity (N=48) | Cholesterol (N=74) |
| Left NBM  FW | **ρ=0.55,p=3.4x10^-7^** | **ρ=0.23,p=0.05** | ρ=-0.13,p=0.27 | ρ=-0.22,p=0.06 | **ρ=0.44,p=0.002** | ρ=-0.08,p=0.58 | ρ=-0.00,p=0.99 |
| Right NBM  FW | **ρ=0.50,p=5.0x10^-6^** | ρ=-0.22,p=0.06 | ρ=0.19,p=0.11 | **ρ=0.33,p=0.004** | **ρ=0.37,p=0.009** | ρ=0.14,p=0.33 | ρ=-0.09,p=0.47 |

BP=Blood Pressure; CRP=C-reactive protein; FW=free-water; HDL=High Density Lipids; NBM=Nucleus of Basal Meynert; Bold values indicate significance at the p<0.05. Units of measurement for BP in mmHg, Triglycerides in mg/dL, homocysteine (mcmol/L), CRP Sensitivity in mg/L and cholesterol in mg/dL.

Supplementary Table 6 Table. Spearman rho correlations among left and right nucleus basalis of Meynert free-water levels, cardiovascular lab panel measures, and blood pressure measurements for *APOE* ε3/4 heterozygous carriers.

|  | Cardiovascular Lab Panel Measures & Blood Pressure Measurements | | | | | | |
| --- | --- | --- | --- | --- | --- | --- | --- |
| Region of Interest | Systolic  BP (N=45) | Diastolic  BP (N=45) | Triglycerides (N=43) | Cholesterol/HDL Ratio (N=43) | Homocysteine (N=28) | CRP High Sensitivity (N=28) | Cholesterol (N=43) |
| Left NBM  FW | **ρ=0.41,p=0.005** | ρ=0.26,p=0.084 | ρ=-0.12,p=0.45 | ρ=-0.06,p=0.72 | ρ=-0.02,p=0.94 | ρ=-0.10,p=0.61 | ρ=0.11,p=0.49 |
| Right NBM  FW | **ρ=0.38,p=0.01** | ρ=-0.21,p=0.16 | ρ=0.08,p=0.51 | ρ=-0.02,p=0.89 | ρ=0.06,p=0.77 | ρ=-0.24,p=0.21 | ρ=-0.04,p=0.81 |

BP=Blood Pressure; CRP=C-reactive protein; FW=free-water; HDL=High Density Lipids; NBM=Nucleus of Basal Meynert; Bold values indicate significance at the p<0.05. Units of measurement for BP in mmHg, Triglycerides in mg/dL, homocysteine (mcmol/L), CRP Sensitivity in mg/L and cholesterol in mg/dL.

Supplementary Table 7 Table. Spearman rho correlations among left and right nucleus basalis of Meynert free-water levels cardiovascular lab panel measures, and blood pressure measurements for *APOE* ε4 homozygous carriers.

|  | Cardiovascular Lab Panel Measures & Blood Pressure Measurements | | | | | | |
| --- | --- | --- | --- | --- | --- | --- | --- |
| Region of Interest | Systolic  BP (N=27) | Diastolic  BP (N=28) | Triglycerides (N=27) | Cholesterol/HDL Ratio (N=27) | Homocysteine (N=19) | CRP High Sensitivity (N=19) | Cholesterol (N=27) |
| Left NBM  FW | ρ=0.23,p=0.25 | ρ=0.07,p=0.74 | ρ=-0.14,p=0.50 | **ρ=-0.40,p=0.04** | **ρ=0.48,p=0.05** | ρ=-0.25,p=0.34 | ρ=-0.07,p=0.73 |
| Right NBM  FW | ρ=0.11,p=0.59 | ρ=-0.11,p=0.56 | ρ=0.13,p=0.51 | ρ=0.23,p=0.25 | ρ=0.44,p=0.08 | ρ=0.04,p=0.89 | ρ=0.10,p=0.63 |

BP=Blood Pressure; CRP=C-reactive protein; FW=free-water; HDL=High Density Lipids; NBM=Nucleus of Basal Meynert; Bold values indicate significance at the p<0.05. Units of measurement for BP in mmHg, Triglycerides in mg/dL, homocysteine (mcmol/L), CRP Sensitivity in mg/L and cholesterol in mg/dL.

Supplementary Text A. Analysis from Regions of Interest sensitive to neurodegenerative changes for Free-water

Anterior Nigra Results

There were no significant main effects of APOE group (p=.696) or CV Risk category (p=.546), and the interaction was also not significant (p=.252). Pairwise comparisons of APOE within levels of CV Risk and vice versa revealed no adjusted p-values less than .05

Left LC to Entorhinal Cortex

A trend toward significance was observed for the main effect of APOE group (F(2,135)=3.05,p=.051,ηp2​=.043), while the main effect of CV Risk (p=.341) and the APOE× CV Risk interaction (p=.285) were not significant. In pairwise comparisons, no contrasts survived Bonferroni correction at Low or High CV Risk.

Right LC to Entorhinal Cortex FW

There were no significant main effects for APOE (p=.529), CV Risk (p=.491), or their interaction (p=.949).

Left Hippocampus FW

The main effects of APOE (p=.166) and CV Risk (p=.444) were not significant. However, the APOE× CV Risk interaction was significant (F(4,135)=2.95,p=.022,η_p_^2^​=.080). In pairwise comparisons, individuals with the ϵ3/ϵ4 genotype in the High CV Risk group had significantly higher FW than their ϵ3/ϵ3 counterparts (Δ=0.056,SE=0.022,p=.031).

Right Hippocampus FW

Main effects of APOE (p=.271) and CV Risk (p=.231) were non-significant, but the APOE× CV Risk interaction showed a trend (F(4,135)=2.14,p=.080,η_p_^2^​=.060).

Supplementary Text B. Analysis from Regions of Interest sensitive to neurodegenerative changes for Free-water corrected FA

Anterior Nigra Results

There was a significant main effect of CV Risk (F(2,135)=6.94,p=.001,η_p_^2^​=.093), but no main effect of APOE (p=.251) or an interaction (p=.300). Pairwise comparisons for CV Risk showed that the High risk group had significantly higher FA than the Low risk group (Δ=0.065,SE=0.021,p=.006), and the Moderate risk group also had higher FA than the Low risk group (Δ=0.048,SE=0.016,p=.012). The High and Moderate risk groups did not differ significantly.

Left LC to Entorhinal Cortex FA

There were no significant main effects for APOE (p=.224) or CV Risk (p=.418), or interaction (p=.085).

Right LC to Entorhinal Cortex FA

All main effects and interactions were non-significant (p>.30)

Left Hippocampus FA

There was a significant main effect of CV Risk (F=4.11,p=.018,η_p_^2^​=.057), but no significant main effect of APOE (p=.160) or an interaction (p=.222). Collapsed across APOE groups, pairwise comparisons showed that the High CV Risk group had significantly higher FA than the Low CV Risk group (Δ=0.027,SE=0.010,p=.016)

Right Hippocampus FA

Main effects of CV Risk (p=.157) and APOE (p=.462), as well as their interaction (p=.349), were not significant.
